# Investigating UAV-Based Applications in Indoor–Outdoor Sports Stadiums and Open-Air Gatherings for Different Interference Conditions beyond 5G Networks

**DOI:** 10.3390/s23156721

**Published:** 2023-07-27

**Authors:** Akhil Gupta, Prakhar Saini, Banala Sharath Teja, Giddaluru Shiva Durgesh, Shourabh Kumar Mishra, Anjani Kumar Yadav, Sudeep Tanwar, Fayez Alqahtani, Maria Simona Raboaca, Wael Said

**Affiliations:** 1 School of Electronics and Electrical Engineering, Lovely Professional University, Phagwara 144001, India; akhil.20239@lpu.co.in (A.G.); prakharsaini2002@gmail.com (P.S.); tejasharath44@gmail.com (B.S.T.); sivadurgesh17@gmail.com (G.S.D.); shourabhmishra1999@gmail.com (S.K.M.); anjani28yadav@gmail.com (A.K.Y.); 2 Department of Computer Science and Engineering, Institute of Technology, Nirma University, Ahmedabad 382481, India; 3 Software Engineering Department, College of Computer and Information Sciences, King Saud University, Riyadh 12372, Saudi Arabia; fhalqahtani@ksu.edu.sa; 4 Doctoral School, University Politehnica of Bucharest, Splaiul Independentei Street, No. 313, 060042 Bucharest, Romania; 5 National Research and Development Institute for Cryogenic and Isotopic Technologies—ICSI Rm. Vâlcea, Uzinei Street, No. 4, Râureni, P.O. Box 7, 240050 Râmnicu Vâlcea, Romania; 6 Computer Science Department, Faculty of Computers and Informatics, Zagazig University, Zagazig 44511, Egypt; wael.mohamed@zu.edu.eg

**Keywords:** UMa, UMi, RMa, LOS, path loss, t-r separation, receive power, human blockage, TX antenna, RX antenna, penetration loss, O2I loss

## Abstract

With the onset of 5G technology, the number of users is increasing drastically. These increased numbers of users demand better service on the network. This study examines the millimeter wave bands working frequencies. Working in the millimeter wave band has the disadvantage of interference. This study aims to analyze the impact of different interference conditions on unmanned aerial vehicle use scenarios, such as open-air gatherings and indoor-outdoor sports stadiums. Performance analysis was carried out in terms of received power and path loss readings.

## 1. Introduction

As technology is developing on a large scale, the 5th generation (5G) is the most advanced technology that can enable wireless communication between humans, sensors, and machines. This rapid evolution upgraded life in instant communication, quick interaction, and good quality of life. The major kits such as millimeter waves and heterogeneous networks lead a straight path in the research of 5G. When the first generation (1G) was introduced in 1979, it was only analog telecommunications, and then an upgradation with text messages came with the name second generation (2G). Now it is time for the fifth generation (5G) with improved data capacity. These 5G and beyond networks are required in urban, rural, and suburban areas. To fulfill the requirement of a good quality network, unmanned aerial vehicles (UAVs) have been used, which temporarily provide a network in regions such as indoor sports stadiums, outdoor sports stadiums, and open area gatherings [1,2].

According to channel measurement results, similar large- and small-scale parameters (SSP) must be obtained for two users with close intervals. It should change accordingly when the user changes or smoothly moves to a different terminal over time. Another effect is called the Doppler shift. It is caused by moving or changing the receiver and transmitter at a time that destroys the measurements of channel parameters. Whenever a change in frequency and the position of the transmitting and receiving antennas occurs, the Doppler shift occurs. Therefore, to eliminate this effect, a new 5G channel model has been introduced, which can handle changes in the parameters of channels or changes in time dimensions [3,4,5].

Millimeter wave is the key to providing more bandwidth, which helps provide high-speed networks such as high-speed downloads. With the inception of the millimeter wave band, the bandwidth of the communication network has increased, which results in a high-speed network, low latency communication, and increased capacity of users. However, the link quality of millimeter waves has faced many propagation issues due to the interference of weather factors such as rain, humidity, and human blockage. Moreover, this challenging issue has been sorted by deploying millimeter wave frequency-based 5G mobile networks in a particular environment and identifying the stable performance of specific areas. Therefore, the millimeter wave frequencies of the propagation channel should be characterized thoroughly. Outdoor measurements have recently confirmed that carrier frequencies at 28 GHz and 38 GHz are giving an optimal performance in particular small areas such as a cell size of 200 m [5]. The channel conditions exist in multiple scenarios such as Urban Macro (UMa), Urban Micro (UMi), and Rural Macro (RMa) [6,7,8].

Due to the increase in the number of users, providing optimal bandwidth has presented an issue. New technology, namely unmanned aerial vehicles (UAVs), has been introduced to resolve this issue. UAVs provide ground users (GUs) with flexible communications between humans and machines. UAVs, also called drones, have mainly improved three useful wireless future phases: improved mobile broadband (eMBB), mobile internet communication (mMTC), and hyper-low latency communication (URLLC). These three aspects explain how important UAVs are in 5G networks. The bandwidth that can be used to supply the wireless communication infrastructure can be licensed or unlicensed. The licensed spectrum functions for UAVs can have several methods, such as cellular bands and satellite technologies. In contrast, unlicensed spectrum bandwidth is owned by numerous parties so that it targets additional situations of interference and less bandwidth availability. Considering the applications of UAVs, recently, UAVs introduced UAV remote sensing technology, and this technology is widely used in military defense, surveying, mapping management, etc. This application is for agricultural monitoring and disaster and emergency response management. UAVs are saving many lives. Furthermore, 5G helps reduce the time between sending and receiving commands sent by GUs. The UAV is a component of an unmanned aircraft system (UAS). This includes the UAV GPS module, ground control module, and the camera set with all the software [9].

There are some interference cases in measuring millimeter wave values, such as O2I (Outdoor to indoor), rain, buildings, trees, vehicles, and human blockages. Outdoor to indoor penetrations will have some interference in the millimeter wave with the change in location and channel. Rain is also one factor or case in which the signals of 5G networks deviate and cause power loss. To solve this, the NYUSIM software values are considered. The rain rate measures 0 mm/h to 150 mm/h in NYUSIM. Buildings, trees, and vehicles will also cause the signal breakdown. Moving vehicles need the ability to mold continuously as per the change of channels. A human blockage is the main issue or interference in the present day as the population is increasing gradually. The signals or the millimeter waves receiving or transmitting may be disturbed when a human blocks the path. However, the interferences are being resolved daily with new techniques [10,11].

Here, the software known as NYUSIM has been used, which contains many channels with different parameters to find the measurements on 5G bandwidths and many more. The software version used is 3.1, an update to the 3.0 version. The major difference compared between the 3.0 and 3.1 versions was the presence of rain rate and human blockage calculators in the new version. NYUSIM is used for millimeter wave and Sub-THz channel simulations. Using the NYUSIM software, different parameters can be found, such as channel parameters, antenna parameters, human blockage parameters, and spatial parameters [12,13].

- Channel Parameters

Channel parameters contain many parameters such as the following scenarios: UMi (urban micro to find the readings in sub-urban areas), UMa (urban macro to find the readings in urban areas), RMa (rural macro to find the readings in rural areas), and InH (indoor to find the readings in indoor or closed areas such as gathering places). In channel parameters, different scenarios are frequencies from 0.5 to 100 GHz. The frequencies considered are 28 GHz, 38 GHz, 60 GHz, and 72 GHz with human interference and rain. The variation in temperature, humidity, distance range (DR), type of environment, and rain rate in mm/h have been considered in channel parameters.

- Antenna Parameters

Antenna parameters contain the parameters for which it can control the antenna locations and the count of antennas required for optimal signals. For example, the number of counts of a transmitter (TX) and receiver (RX) antenna for better measurements of optimal waves and connections can vary.

- Human blockage parameters

Human blockage parameters have been considered due to power loss in the signal due to human interference in the channel. This human interference has been considered practical and has not been considered in an ideal case.

- Spatial Consistency Parameters

Spatial consistency parameters can also be considered. This is the software where the perfect measurements to manage and control the millimeter waves and 5G bandwidth can be found.

### 1.1. Background Survey

This paper discusses the usage of UAVs in indoor-outdoor sports stadiums and open-air gatherings for millimeter wave frequencies of 5G and beyond communication networks. This paper also considers interference factors such as rain, buildings, vegetation, vehicles, and humans. A rigorous background survey has been conducted, and the existing literature has been summarized in Table 1.

In [1], the author outlines the rationale for new millimeter wave cellular systems, methodology, and measurement gear, as well as a range of measurement data demonstrating that 28 and 38 GHz frequencies can be employed when using steerable directional antennas at base stations and mobile devices. In [2], the author examines the channel models used in 5G radio systems. The broad framework for channel models and the key differences between millimeter wave and microwave channel models are also discussed. In [3], the author investigates different channel models created for millimeter wave communication systems using the NYUSIM channel simulator. The created channels were analyzed for carrier frequencies of 28/73 GHz, MIMO antenna configurations from 2 × 2 to 64 × 64, and LOS/NLOS parameters. Based on stochastic geometry, the author develops an analytical model for downlink exposure in massive multiple-input multiple-output (MIMO) antenna networks for 5G. Then, the author analyzes different deployment scenarios of massive MIMO (e.g., cell-free, IoT, etc.). It can also benefit from realistic data representing the transmission gain after deploying massive 5G MIMO antennas into the 5G network [4]. In [5], the author evaluates the performance of the digital beam steering (DBS) precoder in millimeter waves multi-user multiple-input multiple-output (MIMO) systems. Using NYUSIM, realistic statistical features are calculated in 3D. In [6], the author examines how high temperatures, intense humidity, foliage, and more considerable raindrop size impact wireless communication in tropical regions using NYUSIM simulations. In [7], the author proposed a general approach to calculating the per-cell spectral efficiency of millimeter wave multicell single-stream systems. For 5G communications, the author explores the use of SSCM in unlicensed V bands (specifically 60 GHz) while considering both LOS and NLOS conditions. The NYUSIM channel simulator represents the channel characteristics of the 5G backhaul scenario [8]. In [9], the author discusses the use of UAVs in indoor and outdoor sports stadiums, open-air millimeter waves frequencies, and extreme interference factors such as rain, buildings, vegetation, vehicles, and people. Several weather factors are discussed in [10] regarding signal intensity in various settings and circumstances. Based on the NYUSIM simulator, predictions of the channel’s performance are made. Using four frequencies, 30 GHz, 40 GHz, 60 GHz, and 80 GHz, the author evaluated the effectiveness of the channel and chose the best frequency for a tropical setting where rain attenuates between the transmitter and reception antenna. The author presents an analysis of the O2I penetration loss of millimeter waves channels at 28, 38, 60, and 73 GHz operating frequencies for different scenarios: Low loss/high loss and TX/RX antenna HPBW azimuth/elevation 10°/15°. The type of building (standard glass, wood, IRR glass, and concrete) and antenna properties affect channel characteristic O2I penetration loss [11]. In [12], the author compares three 5G channel models, i.e., QuaDRiGa, NYUSIM, and MG5G, from the perspectives of modeling methodologies, parameter settings, and channel simulations. He concludes that NYUSIM gives better results than other channel models and is also more suitable for the RMa scenario. 

In [13], the author demonstrated that these new modeling capabilities reproduce realistic data when implemented in a Monte Carlo manner with NYUSIM 2.0, making it a useful measurement-based channel simulator for designing and evaluating fifth generation and beyond millimeter wave communication systems. In [14], the author created a two-level beamforming architecture for uniform linear arrays that takes advantage of the creation of spatial lobes. Simulations with the channel simulator NYUSIM were used to study the effect of subarray spacing on the spectral efficiency. The findings can be used to create antenna array topologies for 5G wireless systems. Several weather factors are discussed in [15] regarding signal intensity in various settings and circumstances. Based on the NYUSIM simulator, predictions of the channel’s performance are made. Using four frequencies, 30 GHz, 40 GHz, 60 GHz, and 80 GHz, the author evaluated the effectiveness of the channel and chose the best frequency for a tropical setting where rain attenuates between the transmitter and reception antenna. An evaluation of multi-user massive multiple-input multiple-output (MIMO) systems is presented in this paper. The author examines a downlink single-cell scenario that uses linear precoding for zero-forcing (ZF) and conjugate beamforming (CB). A statistical 5G propagation channel was used for this evaluation, developed by NYUSIM [16]. The author performed on [4] simulated spatial channel modeling features for 73 GHz millimeter wave band using NYUSIM. The spatial consistency channel model for moving users and the channel model for static users without consideration of spatial consistency are compared with different channel parameters for LOS and non-LOS (NLOS) environments. Based on stochastic geometry, the author develops an analytical model for downlink exposure in massive multiple-input multiple-output (MIMO) antenna networks for 5G. Then, the author analyzes different deployment scenarios of massive MIMO (e.g., cell-free, IoT, etc.). It can also benefit from realistic data representing the transmission gain after deploying massive 5G MIMO antennas into the 5G network [17]. The author uses NYUSIM software to analyze the performance of MIMO channels at 77 GHz under different configurations. Simulations are conducted in an NLOS environment with MIMO uniform linear arrays at the transmitter and receiver sides [18]. Using the NYUSIM tool [19], the author simulates a 5G channel at the E-band frequency. The urban microcell (UMi) environment was used in this study to assess the effects of massive MIMO and MIMO on LOS and NLOS. In both LOS and NLOS environments, directional and omnidirectional antennas, power delay profiles (PDPs), root mean squares (RMSs) delay spread, and small-scale PDPs were considered. In [20], the author presents a channel model for 5G millimeter wave cellular communication for urban microcells operating at 28 GHz in LOS conditions using multiple antenna elements at the transmitter and receiver. Different parameters affecting the channel have been considered in the simulation using NYUSIM software developed by NYU Wireless. 

The author of [21] created a 3D spatial statistical channel model for millimeter wave and sub-THz frequencies in LOS and NLOS scenarios in an interior office building using comprehensive 28 and 140 GHz observations. In [22], the author investigated NYURay, a 3D millimeter wave and sub-THz ray tracer. This tracer has been calibrated for wireless channel propagation measurements at 28, 73, and 140 GHz in indoor, outdoor, and manufacturing settings. Root mean squares (RMSs) delay spread, and small-scale PDPs were considered. Indonesia’s capital, Jakarta, is a tropical region with high rainfall; therefore, to support the success of initial 5G development planning, it is important to be aware of the channel characteristics over the frequency in Jakarta. Based on simulation results of the NYUSIM channel simulator in [22], the author examines how the characteristics of 5G channels are expressed in the power delay profile (PDP). Using the NYUSIM channel simulator, the author investigates how peripheral variations related to Baghdad city affect millimeter wave transmissions for different frequency bands at millimeter wave. In this study, the diurnal variation in atmospheric conditions limits the performance of millimeter wave transmissions, and critical design insights are pointed out when designing 5G systems [23]. In [24], the author examines millimeter wave communications for 5G. To meet the challenges of millimeter wave communication, architectures and protocols must be redesigned, including integrated circuits and system design, interference management and spatial reuse, antiblockage, and dynamics related to mobility. Current solutions have been reviewed and compared based on effectiveness, efficiency, and complexity. The author explores how 3GPP approaches challenges related to 5G millimeter wave standardization and how solutions can help achieve broader bandwidths and harness some of the inherent benefits of higher-frequency communications [25]. The author discusses several issues that must be resolved to use beamforming for access to millimeter wave frequencies, presents solutions for initial access, and validates them by simulations, showing that millimeter wave frequencies can be used for reliable network access [26]. The author discusses the potential benefits and challenges of the 5G wireless heterogeneous network (HetNet) incorporating massive MIMO and millimeter wave technologies [27]. In [28], the author discusses millimeter wave cellular systems coverage and capacity, emphasizing their key distinguishing characteristics, including the limited scattering nature of the channels and how RF beamforming strategies, such as beam steering, can provide highly directional transmission with minimal hardware complexity. The first performance evaluation of TCP congestion control in next generation millimeter wave networks is presented in [29]. In addition, the framework incorporates detailed models of the millimeter wave channel, beamforming, and tracking algorithms based on real measurements of New York City channels and detailed ray trace analysis.

Furthermore, 5G improves throughput, latency, network reliability, energy efficiency, and connectivity. In addition, the proliferation of smartphones, Internet of Things (IoT) devices, and new multimedia applications have increased the amount of mobile data, which has led to an increase in terahertz technology, communication technology, and 6G wireless communication solutions. Terahertz (THz) technology is expected to play an important role in the development of wireless communication in 6G and beyond with its ability to provide high-speed data transfer and low latency. However, the system faces many challenges, including limitations in internal and external environments due to path loss, reduced access to the environment’s natural process and absorption, and standard processes of 5G and 6G networks that software vulnerabilities can attack. The key to meeting these challenges is using artificial intelligence (AI) to create stronger, more efficient terahertz communication protocols. The scope of related work with the advanced technologies is highlighted in Table 2 [30,31,32,33,34].

### 1.2. Contributions

The millimeter wave band has become prominent with the advent of 5G and beyond communication networks. This study examines the millimeter wave bands working frequencies. The main contribution of this paper is as follows:

- In this paper, we have considered all possible working frequencies of millimeter wave communication networks such as 28 GHz, 38 GHz, 60 GHz, and 72 GHz.
- This work examines the effect of multiple interferences in millimeter wave communication networks such as O2I penetration, rainfall, and human blockage.
- This paper has also worked on UAV-based application use cases scenarios such as indoor–outdoor sports stadiums and open-air gatherings where the need for quality of service is of prime concern.
- In this work, we also analyzed the optimal number of antennae in all possible use case scenarios such as indoor-outdoor sports stadiums and open-air gatherings within different levels of interference conditions.

### 1.3. Organization

The organization of this paper is described as follows: Section 1 gives an insight into the introduction of the 5G and beyond communication networks. Section 2 comprehensively describes scenarios, frequencies, environment, antenna, spatial consistency, and human blockage parameters. The simulation results of the analyzed scenarios and conditions are presented in Section 3. Future perspectives and scope of the research work are depicted in Section 4. Section 5 ends the paper with a conclusion. 

## 2. Millimeter Wave Scenario Parameters

The ultra-wideband (millimeter wave) scenario simulation system NYUSIM allows an accurate model of wireless communication systems. For the millimeter wave scenario, some primary considerations that can be set in NYUSIM are as follows:

- *Carrier frequency:* The frequency at which the signal is transferred is the carrier frequency. Carrier frequencies in millimeter wave systems are typically between 24 and 100 GHz.
- *Bandwidth:* The term “bandwidth” describes the spectrum of frequencies used to transmit the signal. The bandwidth of millimeter wave mm-systems is frequently numerous (up to several gigahertz).
- *Antenna Gain:* An antenna’s gain measures how much it can amplify a signal. Directional antennas are frequently employed in millimeter wave systems to attain high gain.
- *Antenna Bandwidth:* This refers to the antenna beam angular width. Narrow beam widths are preferred in millimeter wave systems to lessen interference.
- *Pathloss model*: The attenuation of the signal as it travels through the environment is determined using this mathematical model. Due to the substantial attenuation brought on by obstacles in millimeter wave systems, the path loss is often much larger than in lower-frequency systems.
- *Antenna Placement:* The performance of millimeter wave systems can be considerably impacted by the positioning of antennas in the surrounding area. To simulate realistic settings, NYUSIM enables the deployment of antennas in particular positions and directions.
- *Scattering Model:* This model is intended to simulate signal scattering caused by environmental barriers. Scattering can result in considerable multipath propagation in millimeter wave systems, which can cause interference and signal deterioration [14,15,16,17].

### 2.1. Urban Micro and Urban Macro Scenario

**Figure 1:**
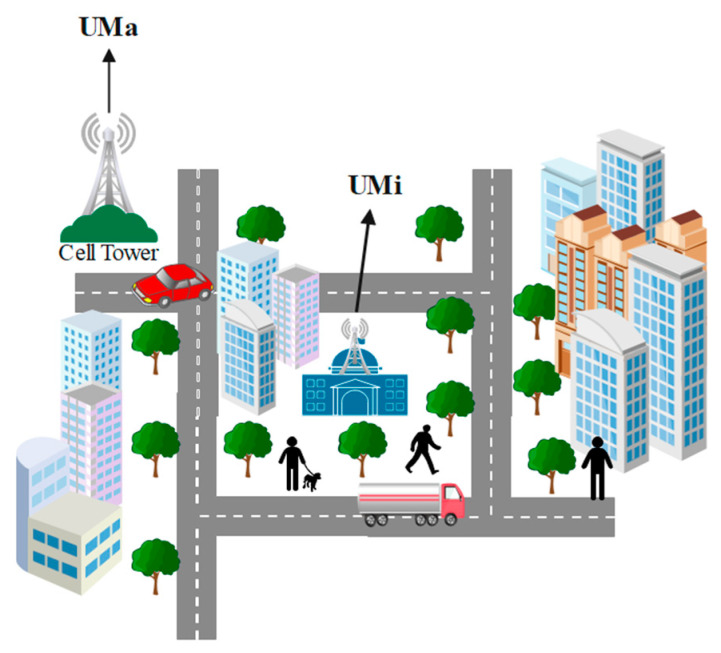
UMa and UMi scenario.

There are different environmental cases or scenarios that are being used to analyze the propagation of the signals, UMa (urban macrocell) and UMi (urban microcell) are among them, as shown in Figure 1. Urban areas contain more traffic, buildings, and people than rural areas. Thus, the propagation of the 5G network will have to face some complex scenarios. To solve these complex problems, microcells contain more antennas in urban areas. As the survey compares, the population in a rural area is less than that in an urban area. As a result, the perimeter of human blockage becomes more crux and interesting, which provides the opportunity for this research paper to redefine the perimeter of human blockage and the study of frequency, environment (outdoor/indoor), rainfall, human blockage, and O2I penetration. Considering the case of indoor stadiums, this gives the insight to determine if the density population is high. Still, the interruption due to buildings, rain, and trees is low, which helps this research in a more classified way in which the work is performed very straightforwardly. The perimeter of path loss and received power vary differently in UMa and UMi cases because of indoor and outdoor factors. Outdoors, the perimeter, such as rain, building interruptions, window interruptions, and human blockages, is at a peak level for 5G network propagation waves. As a result, it produces great differences in an outcome generated through the simulation (from NYUSIM software) [18,19].

### 2.2. Rural Macro Scenario

**Figure 2:**
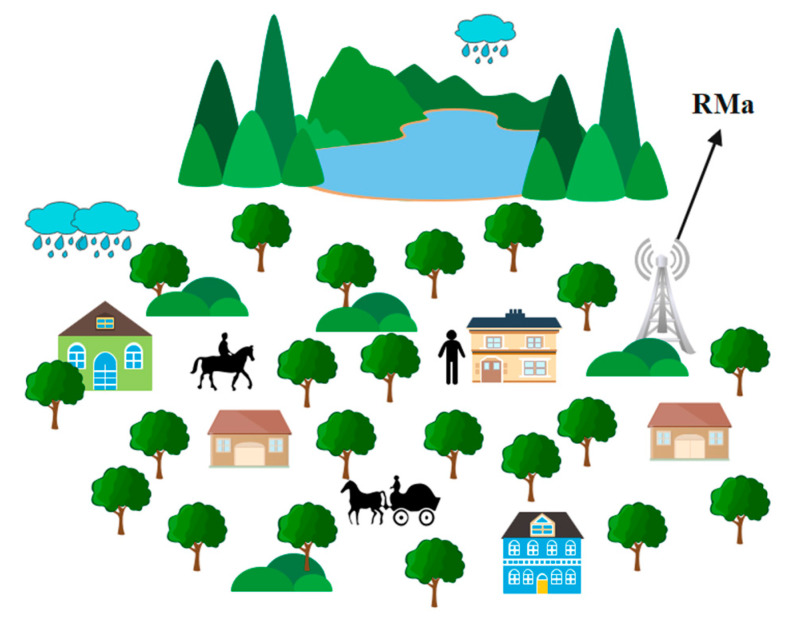
RMa scenario.

This paper analyzes various parameters such as environment (outdoor/indoor), rainfall, human blockage, and O2I penetration, along with various frequencies and different numbers of antennas. RMa is a rural macro scenario, as shown in Figure 2, in which the population is considered low, and the number of buildings and windows (of glass) is considered low. However, the number of trees is taken at high parameters, which fits best for rural areas. The consideration of population is high because of more numbers of agriculture fields, which helps to analyze the accumulated data on human interference and the number of antennas used. As the number of buildings is considered low, several windows (made up of glass) are at minimum consideration, which helps to analyze the parameters such as environment (outdoor/indoor), rainfall, human blockage, and O2I penetration. According to the reference, the amount of research on human blockage has been performed minimally, but here, with the help of the condition of RMa, it is more alarming and makes this paper more eye-opening in the upcoming development on 5G and beyond the network. Hence, a rural microcell is a scenario in the environmental case for 5G network propagation. As rural areas contain less traffic and fewer buildings and people, the usage of propagation is simple compared with urban areas. Thus, fewer macro cells are placed with a smaller number of antennas and sub antennas such as a microcell [10,11].

**Figure 3:**
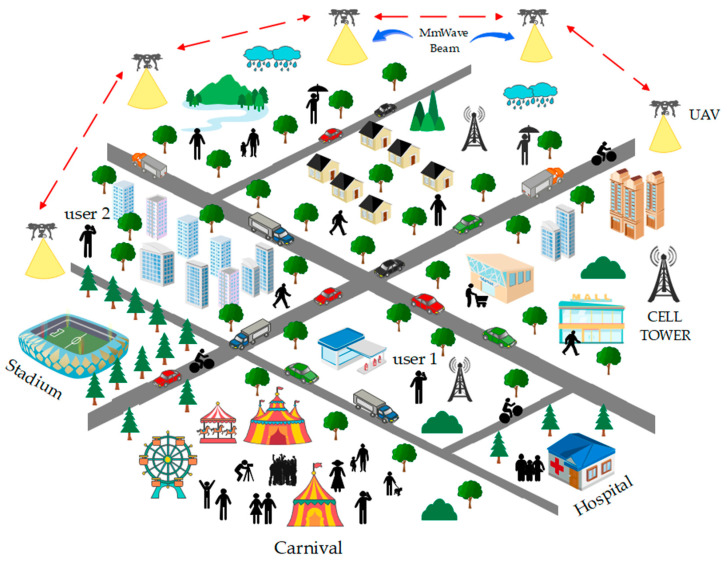
Millimeter wave beamforming using a UAV-based scenario showing interference factors, including rain, buildings, vegetation, vehicles, and people.

Millimeter wave beamforming using the UAV-based scenario shown in Figure 3 depicts users with interference factors such as rain, buildings, vegetation, vehicles, and people, creating a unique interference environment. Rain is one of the most prominent sources of interference, with the high humidity levels in cities leading to more rain and interference. Tall buildings, dense vegetation, and many vehicles also create interference as they can block or weaken the signal. People moving around the city can also cause interference as their bodies can absorb or reflect signals. UAVs can be used to extend the range of the network, providing coverage to areas that are difficult to reach with tower-based networks. Additionally, the use of millimeter wave beamforming technology in UAVs provides several advantages. It allows for higher data rates than traditional terrestrial networks because the signal is focused on a beam and is less affected by interference.

## 3. Simulation Results

This section simulates and analyzes a UAV-based millimeter wave communication network. The analysis has been performed on frequencies such as 28 GHz, 38 GHz, 60 GHz, and 72 GHz. Considering these frequencies, results have been observed in the form of different conditions having all the possible combinations of the human body and rainfall interference that can be applied to different scenarios such as indoor sports stadiums, outdoor sports stadiums, and open area gatherings. This simulation also reflects the optimal number of antennas to provide a better network through better receiver power and path loss. It has been observed that if the signal wave is blocked due to the presence of the human body, the received power decreases and the power loss increases. The simulation parameters used for analyzing the work are mentioned in Table 3.

This work has also been analyzed based on the following different interference conditions: 

*Condition 1 (Human Blockage on and Rain Fall off):* This is the interference condition in which the effect of rainfall is not considered, while the effect from the presence of the human body is considered depending upon the density of the user’s area. Different interference conditions affect the analyzed values of path loss and received power. Since the effect of rain is off in this case and only human blockage is considered, this condition has been used specifically in indoor sports stadiums where there is a huge density of humans and no possibility of rain.

*Condition 2 (Human Blockage off and Rainfall off):* This interference condition has been considered the ideal case in which rainfall and the human body are not considered. This suggests no interruption between t-x and r-x antennas. The maximum value of received power and the minimum value of path loss in this condition are expected. This has minimum interference, so it has considered open area gatherings in rural areas where human participation is significantly less and there is no rainfall interference.

*Condition 3 (Human Blockage on and Rainfall on):* In contrast, considering the interference is in a way keeping the human body and rainfall interference. In this situation, the hindrance is maximum, so path loss would be maximum and received power would be minimum and considered a worst-case scenario. This is also considered in urban areas such as open sports stadiums with maximum human blockage and rain.

*Condition 4 (Human Blockage off and Rainfall on):* The main study of propagation waves is to observe how they affect rain and the areas where human density is lowest, such as rural areas, and can be considered as open area gatherings in the rain where human density is low. In this condition, there is only rainfall interference and no human interference.

### 3.1. Indoor Sports Stadium

Nowadays, there is a huge requirement for better signals and good communication speed. This requirement becomes difficult to fulfill in the case of too many users in a particular region. Therefore, to solve this problem, some results have been researched and have shown the best number of antennas required to solve this problem. Indoor sports stadiums include a huge density of humans, so it is considered human blockage here. There is no scope for rain in an indoor sports stadium [9,20].

Table 4 shows the optimal number of antennas for indoor sports stadiums. In an indoor sports stadium, the work is performed to analyze the optimal number of antennas required for better quality of services at different working frequencies with different interference conditions. This analysis is performed based on factors such as received power and path loss.

The analysis is performed regarding an optimal number of antennas for better-received power and path loss. If a human density is much less than it is being reflected by Condition 2. After the simulation work, it is concluded from Table 4 that for a 28 GHz working frequency, the optimal number of antennas for Conditions 1 and 2 is **2** in case of both better-received power and path loss. Similarly, if the millimeter wave is of 38 GHz frequency, the optimal number of antennas required for better-received power, and path loss is **4** for Condition 1 and 2 for Condition 2. With the signal having a frequency of 60 GHz, the optimal number of antennas required for better-received power and path loss is **4** for Condition 1, and for Condition 2 it is **4** for received power and 2 for path loss.

### 3.2. Outdoor Sports Stadium

In this case, both possibilities with human and rain interference are considered. Table 4 shows the significant and optimal number of antennas for factors such as received power and path loss. This varies with the amount of human density and the amount of rainfall [6,10,21].

Table 5 reflects the number of optimal antennas for better-received power and path loss concerning the different millimeter wave frequencies. Considering the simulation results, it can be concluded that the number of optimal antennas at 28 GHz frequency is **2** for all conditions for both received power and path loss. Similarly, in the case of 38 GHz frequency and received optimal power number of antennas for Condition 1 is **4**; for Condition 2 it is **2**; for Condition 3 it is **4**; and for Condition 4 it is 4. Similarly, for path loss, an optimal number of antennas for 38 GHz is 2 for Conditions 1 and 2, and several antennas are **4** for Condition 3, and **2** for Condition 4. Similarly, for millimeter wave frequency, 60 GHz optimal number of antennas for better-received power is **4**, **2**, **4**, **4** for Conditions 1, 2, 3, 4, respectively, and for better path loss, the optimal number of antennas is **4**, **2**, **4**, **4** for Conditions 1, 2, 3, 4, respectively. If the millimeter wave frequency is 72 GHz, then the optimal number of antennas for better-received power is **8**, **4**, **8**, **8** for Conditions 1, 2, 3, 4, respectively, and for better path loss the optimal number of antennas is **8**, **2**, **8**, **8** for Conditions 1, 2, 3, 4, respectively.

### 3.3. Open Area Gatherings

Open area gatherings such as rallies, functions, and parties are also important to consider. Specifically, we consider rural gatherings such as rallies and parties. The possibility of having all possible cases of human and rain interferences is in this application [22,23].

Table 6 significantly reflects the efficient number of antennas used in open area gatherings for better-received power and path loss. Human density is less, so the number of antennas in all cases has been observed to be less compared to Table 4 and Table 5. Considering all the simulation results, it has been observed that the number of optimal antennas for better-received power and path loss in the case of millimeter wave with the frequency of 28 GHz is **2** for all Conditions 1, 2, 3, 4. Similarly, in the case of 38 GHz frequency and received power, the optimal number of antennas for Condition 1 is **2**; for Condition 2 it is **2**; for Condition 3 is **4**; and for Condition 4 is **2**. Similarly, for path loss, the optimal number of antennas for 38 GHz is **2** for Conditions 1 and 2, and the number of antennas is **2** for Condition 3, and **2** for Condition 4. Similarly, for millimeter wave frequency, 60 GHz optimal number of antennas for better-received power is **4**, **2**, **4**, **2** for Conditions 1, 2, 3, 4, respectively, and for better path loss, the optimal number of antennas is **4**, **2**, **4**, **4** for Conditions 1, 2, 3, 4, respectively. If the millimeter wave frequency is 72 GHz, then the optimal number of antennas for better-received power is **4**, **2**, **4**, **4** for Conditions 1, 2, 3, 4, respectively, and for better path loss the optimal number of antennas is **4**, **4**, **4**, **4** for Conditions 1, 2, 3, 4, respectively.

### 3.4. Optimal Solutions for Different Scenarios of Millimeter Wave UAV-Based Networks

**Figure 4:**
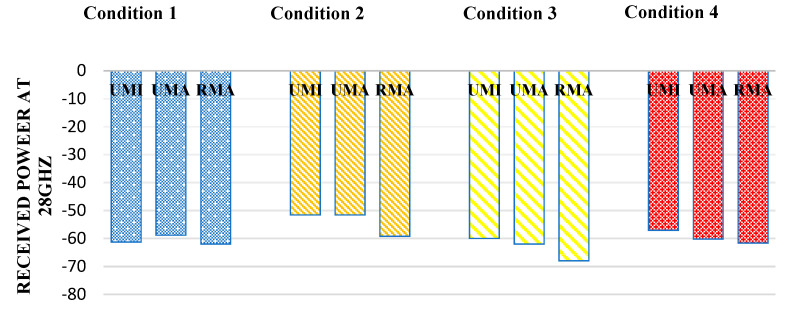
Received power at 28 GHz for different interference conditions in different scenarios such as UMI-indoor sports stadium, UMA-outdoor sports stadium, and RMA-open gatherings.

Figure 4 shows the received power at 28 GHz frequency. Some examples have been considered indoor sports stadiums. For indoor sports stadiums, the condition of no rain and human blockage interference is considered the most suitable. Following it, the received power in this case has been observed as −61.27 watts. The best result has been observed when there is no human blockage and no rain, having received power in the case of an indoor sports stadium is −51.545 watts, and the minimum received power has been observed when both human blockage interference and rain is −59.99 watts. Thus, overall received power in the case of indoor sports stadiums has decreased by 16.38% while considering human blockage and rain interference. Similarly, in the case of outdoor sports stadiums, the received power decreased by 20.27% by considering the worst-case scenario compared to the ideal situation. Similarly, in the case of open gatherings, this value has decreased by 14.8% in the worst-case scenario.

**Figure 5:**
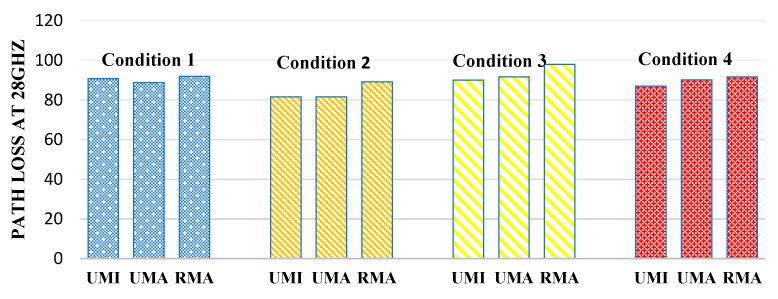
Path loss at 28 GHz for different interference conditions in different scenarios such as UMI-indoor sports stadium, UMA-outdoor sports stadium, and RMA-open gatherings.

Figure 5 defines the path loss at 28 GHz. In the case of an indoor sports stadium, the path loss is minimum when both human blockage and rain interference have not been taken and will be considered an ideal case. Therefore, in the case of indoor sports stadiums, path loss in the worst-case scenario (when there is a human blockage and rain interference is there) has been observed to increase by 10.3%. Similarly, in the case of an outdoor sports stadium, path loss increases by 12.41% in the worst case concerning the ideal case. Similarly, in the case of open gatherings, path loss was observed to increase by 9.82%.

**Figure 6:**
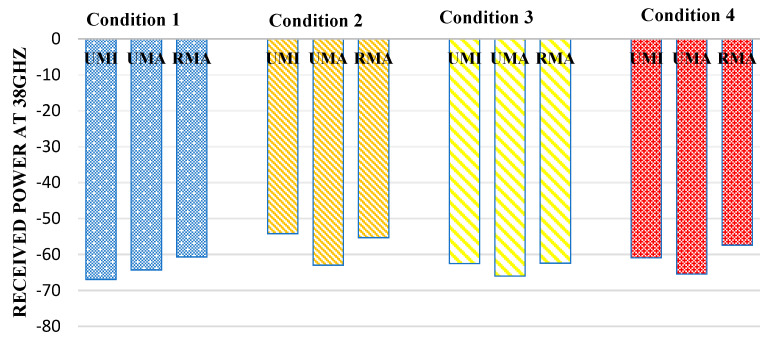
Received power at 38 GHz for different interference conditions in different scenarios such as UMI-indoor sports stadium, UMA-outdoor sports stadium, and RMA-open gatherings.

Figure 6 reflects the received power at 38 GHz. In indoor sports stadiums, the received power is maximum when both human blockage and rain interference are not featured and will be considered an ideal case. Thus, in the case of an indoor sports stadium, the path received power in the worst-case scenario (when human blockage interference and rain is featured) has been observed to decrease by 15.3%. Similarly, in the case of outdoor sports stadiums, received power decreases by 4.8% in the worst case concerning the ideal case. Similarly, in the case of RMA, the received power was observed to decrease by 12.8%.

**Figure 7:**
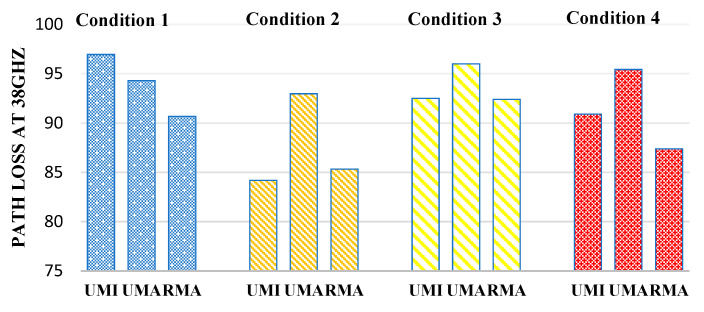
Path loss at 38 GHz for different interference conditions in different scenarios such as UMI-indoor sports stadium, UMA-outdoor sports stadium, and RMA-open gatherings.

Figure 7 defines the path loss at 38 GHz. In the case of indoor sports stadiums, the path loss is minimum when both human blockage and rain have not been taken and will be considered an ideal case. Therefore, in the case of indoor sports stadiums, the path loss in the worst-case scenario (when there is a human blockage and rain interference is there) has been observed to increase by 9.8%. Similarly, in the case of outdoor sports, stadium path loss increases by 3.25% in the worst case concerning the ideal case. Similarly, in the case of open gatherings, path loss was observed to increase by 8.3%.

**Figure 8:**
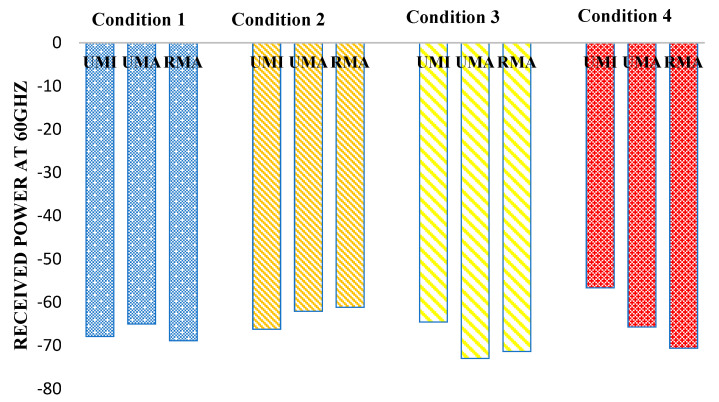
Received power at 60 GHz for different interference conditions in different scenarios such as UMI-indoor sports stadium, UMA-outdoor sports stadium, and RMA-open gatherings.

Figure 8 reflects the received power at 60 GHz. In indoor sports stadiums, the received power is maximum when both human blockage and rain interference are not featured and will be considered an ideal case. Thus, in the case of an indoor sports stadium, the path received power in the worst-case scenario (when human blockage interference and rain is featured) has been observed to decrease by 2.6%. Similarly, in the case of outdoor sports stadiums, received power decreases by 17.6% in the worst case concerning the ideal case. Similarly, in the case of open gatherings, received power was observed to decrease by 16.6%.

**Figure 9:**
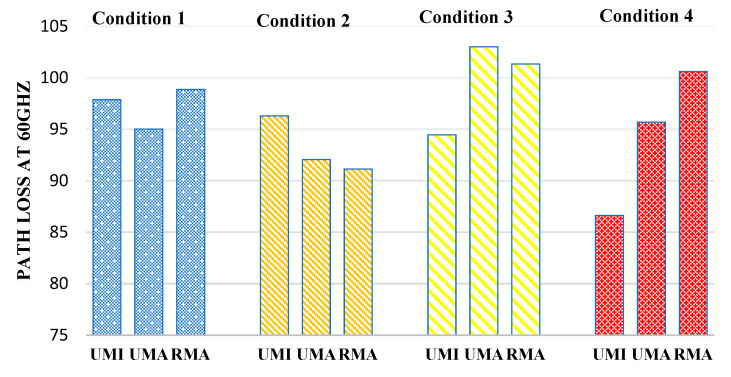
Path loss at 60 GHz for different interference conditions in different scenarios such as UMI-indoor sports stadium, UMA-outdoor sports stadium, and RMA-open gatherings.

Figure 9 defines the path loss at 60 GHz. In the case of indoor sports stadiums, the path loss is minimum when both human blockage and rain have not been taken and will be considered an ideal case. Therefore, in the case of an indoor sports stadium, the path loss in the worst-case scenario (when there is a human blockage and rain interference) has been observed to increase by 1.9%. Similarly, in the case of outdoor sports, stadium path loss increases by 11.8% in the worst case concerning the ideal case. Similarly, in the case of open gatherings, path loss was observed to increase by 11.17%.

**Figure 10:**
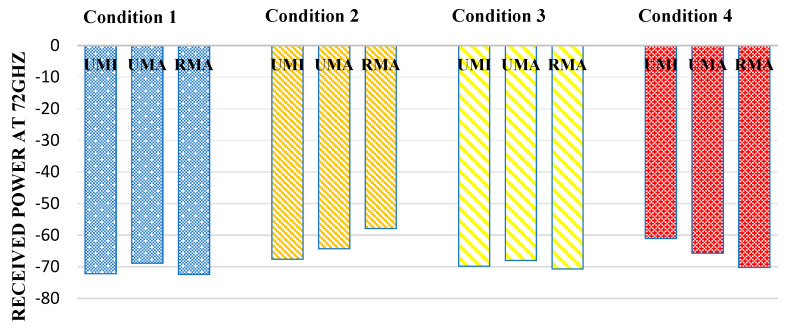
Received power at 72 GHz for different interference conditions in different scenarios such as UMI-indoor sports stadium, UMA-outdoor sports stadium, and RMA-open gatherings.

Figure 10 reflects the received power at 72 GHz. In the case of an indoor sports stadium, the received power is maximum when both human blockage and rain interference are not featured and will be considered an ideal case. Thus, in the case of an indoor sports stadium, receiving power in the worst-case scenario (when human blockage interference and rain is featured) has been observed to decrease by 3.33%, concerning the ideal case. Similarly, received power decreases by 5.7% in an outdoor sports stadium. Similarly, in the case of open area gathering, received power was observed to decrease by 22.17%.

**Figure 11:**
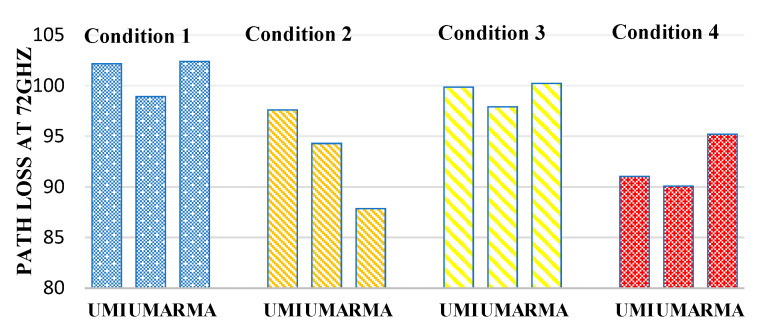
Path loss at 72 GHz for different interference conditions in different scenarios such as UMI-indoor sports stadium, UMA-outdoor sports stadium, and RMA-open gatherings.

Figure 11 defines the path loss at 72 GHz. In the case of an indoor sports stadium, the path loss is minimum when both human blockage and rain have not been taken and will be considered an ideal case. Therefore, in the case of an indoor sports stadium, the path loss in the worst-case scenario (when there is a human blockage and rain interference is there) has been observed to increase by 2.3%. Similarly, in the case of outdoor sports, stadium path loss increases by 3.82% in the worst case concerning the ideal case. Similarly, in the case of open gatherings, path loss has been observed to increase by 14.05%.

## 4. Future Scope

**Figure 12:**
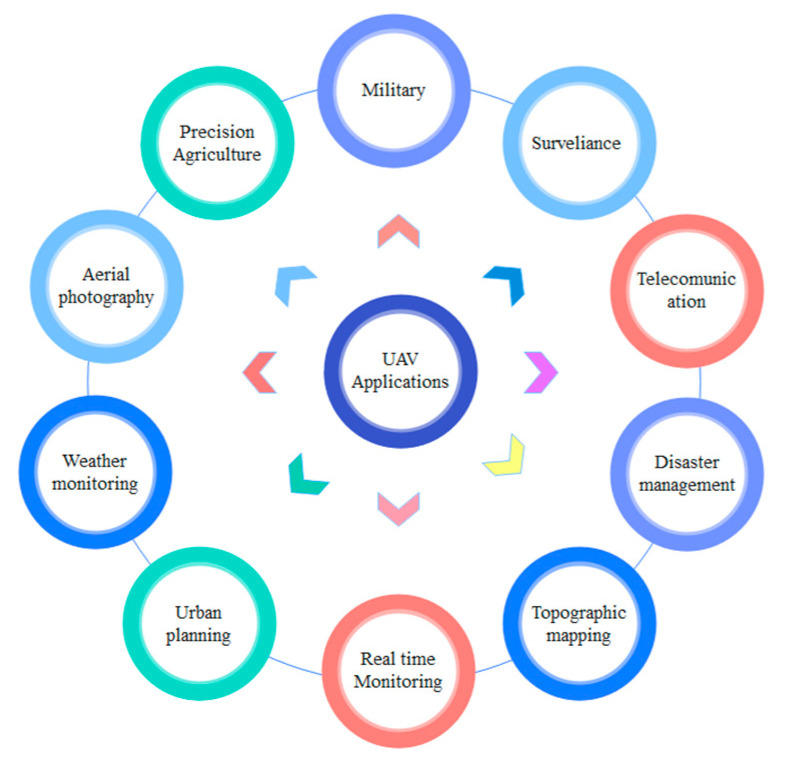
UAV applications.

For a long time, there has been speculation on how 5G technology will be used. It is asserted that 5G will permit further advancements in smart cities, automated vehicles, digital business 4.0, and other areas, and will revolutionize several marketplaces. The most resilient network can be achieved by combining millimeter wave with femtocells and large MIMO, two other symbiotic technologies. This is largely due to the newest advancements and technology incorporated in the 5G system this year, where telecom providers would theoretically reap more benefits from their significant investments. As a result, smartphone vendors will be able to produce more affordable devices, increasing customer demand and resulting in network operators spending less on infrastructure. Mobile broadband advancements also lower power consumption. There are many prospects to find cutting-edge methods for handling networks thanks to the 5G infrastructure. Network slicing, which enables a single physical network to serve many virtual networks with different functionality and features, is born from this. In the chosen example, one network slice would offer high-speed mobile access on the same infrastructure, while another may result in lower network use for the 5G link level. With the help of 5G technology, different networks can frequently be provided to clients and market segments using the same network. With such a significant influence, 5G technologies would increase the financial potential for future creative business structures. UAVs have also been used in a variety of applications, including military, construction, image and video mapping, medical, search and rescue, package delivery, reconnaissance, telecommunication, surveillance, precision agriculture, wireless communication, and weather monitoring. There are several applications with the use of UAVs, and they are depicted in Figure 12 [24,25,26,27,28].

## 5. Conclusions

In conclusion, 5G and beyond communication networks purely focus on increasing the quality of the service of the network. For better service quality, interference conditions need to be monitored and optimal solutions need to be provided. In this paper, O2I penetration loss is considered in all possible cases. However, there have been different scenarios with changing interference properties in terms of the presence of human and rain interference. Considering these interferences, four different interference conditions were considered. The analysis was also made with different millimeter wave frequencies such as 28 GHz, 38 GHz, 60 GHz, and 72 GHz. This work also concluded the optimal number of antennas for better-received power and path loss under different conditions for different millimeter wave frequencies. By using the analysis performed under different conditions, optimal simulation conditions are proposed for indoor sports stadiums, outdoor sports stadiums, and open area gatherings regarding received power and path loss. This paper also reflects the increase and decrease in the percentage of received power and path loss for the ideal case.

## Figures and Tables

**Table 1:** Background survey on the interference factors in different scenarios.

Ref.	Frequency	Environment Outdoor	Environment Indoor	Rainfall	Human Blockage	O2I Penetration	UMI	UMA	RMA
[1]	28 GHz, 38 GHz	✓	✓	✗	✗	✗	✗	✗	✗
[2]	28 GHz, 86 GHZ	✓	✓	✓	✗	✓	✓	✓	✗
[6]	38 GHz	✓	✗	✓	✗	✗	✓	✗	✗
[7]	28 GHz, 60 GHz, 73 GHz	✗	✓	✓	✗	✗	✓	✗	✗
[12]	28 GHz, 73 GHz	✓	✓	✗	✗	✗	✓	✓	✓
[13]	28 GHz, 73 GHz, 140 GHz	✓	✓	✗	✗	✗	✓	✗	✗
[14]	73 GHz	✓	✓	✗	✓	✓	✓	✗	✗
[15]	28 GHz, 140 GHz	✗	✓	✗	✗	✓	✗	✗	✗
[16]	28 GHz, 38 GHz, 60 GHz	✓	✓	✓	✗	✓	✓	✓	✓
[Proposed]	28 GHz, 38 GHz, 60 GHz, 72 GHz	✓	✓	✓	✓	✓	✓	✓	✓

✓ represents the analysis of the mentioned cases in the cited references, while ✗ represents that the mentioned cases are not analyzed in the cited references.

**Table 2:** Scope of related work with the advanced technologies.

References	Technology	Concern	Cause
[30]	Terahertz (THz) waves	Physical-layer security in terahertz wireless networks	Interferences in frequencies
[31]	Internet-of-Things (IoT), cyber–physical systems (CPSs)	Increasing need for communication has led to an increase in security concerns	Latency and Interferences in frequencies
[32]	Terahertz (THz) waves	Use of the highly directional THz waves, eavesdropping attacks on the transmission system can be theoretically prevented.	Higher data transmit, terahertz accelerates 5G towards 6G
[33]	Terahertz (THz) waves, artificial intelligence (AI)	Use of AI in THz, THz role in development of 6G	Interferences, terahertz accelerates 5G towards 6G
[34]	5G and 6G	Network Privacy and Security Issues in 5G and 6G, AI based security in 5G and 6G, security protocols.	Understanding 5G and 6G security issues and assisting 5G towards 6G

**Table 3:** Simulation parameters.

Simulation Parameters	Values
Scenarios	UMi, RMa, UMa
Frequencies	28 GHz, 38 GHz, 60 GHz, 72 GHz
Radio Frequency Bandwidth (0–800 MHz)	800 MHz
Distance Ranging Option	Standard (10–500 m)
Environment	LOS, NLOS
Transmitter to Receiver Separation Distance	10–50 m
Transmitter Power (0–50 dBm)	30 dBm
Base Station Height	3 m
User Terminal Height	1.5 m
The number of Receiver Locations	1
Barometric Pressure	1013.25 mbar
Humidity (0–100%)	50%
Temperature	20 °C
Polarization	Co-Pol
Rain or Precipitation Rate in mm/h	0 mm/h and 150 mm/h
Foliage Loss	No
Distance Within Foliage	0 m
Foliage Attenuation	0.4 dB/m
Outdoor to Indoor (O2I) Penetration Loss	No
O2I Loss Type	Low Loss
Transmission Array Type	ULA
Receiver Array Type	ULA
Number of Transmission Antenna Elements Nt	2, 4, 8, 16
The number of Receiver Antenna Elements Nr	2, 4, 8, 16
Transmission Antenna Spacing (in wavelength, 0.1–100)	0.5
Receiver Antenna Spacing (in wavelength, 0.1–100)	0.5
The number of Transmission Antenna Elements Per Row Wt	1
The number of Receiver Antenna Elements Per Row Wr	1
Transmission Antenna Azimuth HPBW	10 degrees
Receiver Antenna Azimuth HPBW	10 degrees
Transmission Antenna Elevation HPBW	10 degrees
Receiver Antenna Elevation HPBW	10 degrees
Correlation Distance of Shadow Fading	10 m
Update Distance	1 m
Correlation Distance of LOS/NLOS Condition	15 m
Moving Direction	45 degrees
Moving Distance	40 m
User Track Type	Linear
User Velocity	1 m/s
Side Length (Only for Hexagon track)	10 m
Segment Transitions	Yes
Orientation (Only for Hexagon track)	Clockwise
Human Blockage	ON, OFF
Trans. Rate from Unshadow to Decay	0.2/s
Trans. Rate from Decay to Shadow	8.1/s
Trans. Rate from Shadow to Rise	7.8/s
Trans. Rate from Rise to Unshadow	6.7/s
Default Settings for Human Blockage	No
Mean Attenuation	14.4 dB

**Table 4:** The optimal number of antennas for indoor sports stadium.

	Received Power		Path Loss	
Frequency	Condition 1	Condition 2	Condition 1	Condition 2
28	2	2	2	2
38	4	2	4	2
60	4	4	4	2
72	16	4	16	4

**Table 5:** The optimal number of antennas for outdoor sports stadiums.

Received Power					Path Loss			
Frequency	Condition 1	Condition 2	Condition 3	Condition 4	Condition 1	Condition 2	Condition 3	Condition 4
28	2	2	2	2	2	2	2	2
38	4	2	4	2	2	2	4	2
60	4	2	4	4	4	2	4	4
72	8	4	8	8	8	2	8	8

**Table 6:** The optimal number of antennas for open-air gatherings.

Received Power					Path Loss			
Frequency	Condition 1	Condition 2	Condition 3	Condition 4	Condition 1	Condition 2	Condition 3	Condition 4
28	2	2	2	2	2	2	2	2
38	2	2	4	2	2	2	2	2
60	4	2	4	2	4	2	4	4
72	4	2	4	4	4	4	4	4

## Data Availability

Not applicable.

## Abbreviations

UMa	Urban Macrocell
UMi	Urban Microcell
RMa	Rural Macrocell
SBS	Special broadcasting service
GHz	Giga Hertz (unit of frequency)
MIMO	Multiple Input Multiple Output
TX	Transmitter antenna
RX	Receiver antenna
MM	Millimeter
LOS	Loss of sight
O2I	Outdoor to indoor
5G	5th generation
2G	Second generation
V2V	Vehicle to vehicle
HST	High speed train
UAV	Unmanned aerial vehicles
eMBB	Improved mobile broadband
eMTC	Mobile internet communication
URLLC	Ultra reliable low latency communication
GPS	Global positioning system
DR	Distance range
